# Investigation of the Effects of Violence Experience During Political Demonstrations

**DOI:** 10.5964/ejop.v16i3.1991

**Published:** 2020-08-31

**Authors:** Mübeccel Yeniada Kırseven, Sedat Işıklı

**Affiliations:** a Department of Psychology, Hacettepe University, Ankara, Turkey; London School of Economics, London, United Kingdom

**Keywords:** posttraumatic stress, posttraumatic growth, political violence

## Abstract

In this study, predictors of post-traumatic stress symptom levels (PTSSL) and post-traumatic growth levels (PTGL) resulting from the experience of violence were investigated. The sample of the study consisted of 514 Gezi Park demonstrators. Participants completed measures assessing stress symptoms, post-traumatic growth, social support and beliefs about the world as well as the open-ended event specific questions. Results showed that being politically active, psychologically prepared and experiencing mild levels of violence were related with decreased PTSSL individually but not in combination as the literature suggested. The two hierarchical regression analyses showed that: (1) PTSSL were predicted by violence exposure levels, perceived social support from significant others and “randomness” and “self-worth” beliefs about the world; (2) PTGL were predicted by violence exposure levels, total amount of time spent at the demonstrations and “benevolence” and “justice” beliefs about the world. These findings suggest that psychological preparedness might be an important variable in violence experience regarding human masses. Also, violence exposure levels and duration of participation seems to be important event- specific variables. Lastly, political activism needs to be more precisely operationalized and measured in future studies.

Traumatic events differ from other experiences in regard to their unpredictability, uncontrollability, vulnerability, and potential changes in beliefs on the world and life (Saari & Silver, 2005). Research shows that post-traumatic stress disorder (PTSD) is more frequent in the case of human-made disasters than natural disasters (Kessler, Sonnega, Bromet, Hughes, & Nelson, 1995; Lee & Young, 2001). About 30% to 50% of human rights abuses survivors suffer from PTSD (Kaminer, Stein, Mbanga, & Zungu-Dirwayi, 2001; Pillay, 2000; Steel et al., 2009). Moreover, PTSD after human-made disasters is emphasized to be more severe and longer lasting than natural disasters (American Psychiatric Association, 1994), besides the destructive effects of the disaster on survivors as well as their loved ones and witnesses (Breslau & Davis, 1987). The severity of trauma is related to the existence and severity of physical injury, which are due to the traumatic event (North et al., 1999). For example, torture survivors with persistent physical damage, like burns or injury scars, are likely to have higher psychopathology levels (Paker, Paker, & Yüksel, 1992).

Contrary to expectations, results have shown that although both involve suffering and decline in functionality, symptoms in human rights abuses survivors do not fully coincide with PTSD symptoms (Hernández, 2002), which seems to support a previous claim about PTSD not being as prevalent as it was thought to be (Yehuda & McFarlane, 1995). Moreover, it has also been suggested that traumatic events are not only sources of distress, but also an opportunity for growth, discussed as post-traumatic growth (PTG; Tedeschi, 1999). PTG is identified by major and beneficial changes in the cognitive and emotional worldview of survivors, which could also have behavioral effects (Tedeschi, Park, & Calhoun, 1998). It has been claimed that post-traumatic stress and growth can be present simultaneously (Tedeschi & Calhoun, 1996). PTG is seen as the result of survivors’ coping, indicating a more positive level of functionality than before the traumatic event (Tedeschi & Calhoun, 2004). For PTG to arise, distress and suffering are found to be necessary (Calhoun, & Tedeschi, 1998), alongside a higher threat felt during the traumatic event (Calhoun & Tedeschi, 2006). Studies have reported five domains in which PTG has been observed: (1) changes in interpersonal relations, (2) changes in self-perception, (3) realizing the value of life, (4) realizing new opportunities, and (5) changes in the belief system (Tedeschi & Calhoun, 1996). Studies have also shown PTG to be related with the type of traumatic event experienced (Bellizzi, 2004; Karanci et al., 2012).

Compared with individual traumas, *collective traumas* include higher social support, social sharing, social inclusion, and rituals that enhance social inclusion, all of which strengthen people’s beliefs about their coping abilities (Páez, Basabe, Ubillos, & Gonzalez-Castro, 2007). Collective traumas attract less attention than personal traumas (Lifton, 2005); however, wars have been the most frequently studied topic since they are human-made collective disasters (Luszczynska, Benight, & Cieslak, 2009).

As a form of collective trauma, *political traumas* was defined as occurring due to politically motivated human behavior, which might have political consequences and have effects on people’s collectives, destruction, and violence (Vertzberger, 1997). Political traumas were centered upon events that include human rights violations and violence, such as war (Steel et al., 2009), migration (Shannon et al., 2015), and torture (Kaminer, Stein, Mbanga, & Zungu-Dirwayi, 2001). These collectively experienced traumas were referred to as having a potential to not only effect but also create new communities (Erikson & Yule, 1994). With respect to this collectivism, people are able to cope better with the adverse effects of trauma (Vertzberger, 1997). Indeed, there are claims that traumatized communities are better able to withstand stress or react positively (Suedfeld, 1997).

Political violence is mentioned to be the core element of political trauma. Political violence was found to be related with people’s mental (Başoğlu et al., 2005; Steel et al., 2009) and physical health (Başoğlu et al., 2005). For example, after the political tension and violence in 1980, suicidal behavior rates in Sri Lanka rose from 6.5% in 1950 to 47.3% in 1995 (Marecek, 1998). In Turkey, many studies on political violence focused on torture. For instance, exposure to prolonged torture has been related with elevated symptoms of PTSD, depression, and anxiety, but at a moderate level (Başoğlu et al., 1994). Moreover, researchers proposed that preliminary information and preparedness for torture, commitment to a cause, immunity against traumatic stress, and social support might have a protective role against PTSD among torture survivors (Başoğlu et al., 1994, 1997; Paker, 2000).

Political activism was also found to be related with psychological preparedness for torture and decreased PTSD symptoms (Başoğlu et al., 1997). Political activism provides a subjective meaning to trauma, and psychological preparedness could be related with this meaning (Başoğlu et al., 1997; Paker, 2000). Meanwhile, strong ideological commitment mediates between political violence and psychological wellbeing (Muldoon, Schmid, & Downes, 2009). In terms of psychopathologies, subjective meaning is an important predictor, whereas weakness in that meaning, severe violence, and captivity were related with PTSD symptoms (Paker, 2000). After a violent experience, a non-activist group’s political commitment may increase (Paker, 2000).

## World Assumptions

First systematically studied by Janoff-Bulman (1989), world assumptions refer to the unquestioned assumptions of people on themselves and the world. Traumatic events cause people who assume that the world is predictable and safe to question their beliefs and realize their vulnerability. According to Janoff-Bulman (1989), people have three fundamental assumptions: (1) the overall benevolence of the world, (2) the meaningfulness of the world, and (3) self-worthiness. The two sub-assumptions of the *benevolence of the world* refer to the benevolence of the world as an entity, including the benevolence of the people in it. The *meaningfulness of the world* contains justice, controllability, and randomness as sub-assumptions. Lastly, *self-worthiness* comprises self-worth, self-control, and luck (Janoff-Bulman, 1989). After a traumatic event, these assumptions are shattered; acknowledging their vulnerability, survivors are believed to question their positive beliefs of their self and the world (Janoff-Bulman, 1989). In the aftermath of trauma, these assumptions are rebuilt, forming new assumptions that integrate the traumatic experience in lieu of previous assumptions (Janoff-Bulman, 1989, 1992). Janoff-Bulman (1992) further reported that after natural disasters, the assumptions benevolence of the world, randomness, and control are affected the most, whereas after human-made disasters, the assumptions benevolence of the world and the people and self-worthiness are affected the most. Meanwhile, research conducted with tsunami survivors revealed changes in their justice assumptions to be related to elevated PTSD symptoms and decreased life quality (Nygaard & Heir, 2012).

## Social Support

Throughout literature, “perceived” social support has gained increased attention (Eker, Arkar, & Yaldız, 2001; Lazarus, 1990; Yap & Devilly, 2004) compared to “received” social support, which are the two types of social support suggested (Heller, Swindle, & Dusenbury, 1986). Perceived social support protects trauma survivors against depression, anxiety, and stress; whereas its deficiency has been related to elevated distress and plays an important role in trauma symptomatology (Yap & Devilly, 2004). Moreover, perceived social support has an effect on the relationship between the traumatic event and post-traumatic stress, buffering the effects of the traumatic event, although the contribution of social support and its relation with other variables remain unexplained (Haden, Scarpa, Jones, & Ollendick, 2007).

In collective traumas, social support is an important buffer at all societal levels (Hoffman & Kruczek, 2011). A study on political violence revealed that apart from the importance of social support, the individual’s level of satisfaction with the perceived social support buffers the impacts of political violence on mental health (Punamäki et al., 2005). Moreover, studies suggest that collective traumas facilitate higher social support and broaden coping opportunities (Luszczynska, Benight, & Cieslak, 2009). However, there is still controversy regarding findings on the buffering effects of social support on mental health after collective traumas (Grills-Taquechel, Littleton, & Axsom, 2011). For example, a study on the 9/11 attacks found no relationship between social support and physical and mental health (Adams, Boscarino, & Galea, 2006).

## Gezi Park Demonstrations as a Traumatic Experience

The *Gezi Park demonstrations* began as an ordinary environmental protest and spread all over Turkey, becoming a social movement with the active participation of large groups of citizens. Security forces’ use of deterrents, such as pepper gas, batons, rubber bullets, and pressurized water, to disperse the crowds shifted the environmental focus of the demonstrations to an anti-government stance. Amid the demonstrations, the Foundation for Political, Economic and Social Research (SETA; Ete & Taştan, 2013) conducted and published research to bring the misinformation to light. In addition, the Human Rights and Equality Institution of Turkey (TİHK) published a report based on data from the Ministry of the Interior indicating that from May 28 to September 6, 2013, 5,532 demonstrations had been held in 80 cities, attended by approximately 3,611,208 people (2013). Most of the demonstrators were aged between 15 and 29 years, and no gender disparity was found (TİHK, 2014; Ete & Taştan, 2013). According to the Turkish Medical Association (TTB, 2013), 6 civilians and 2 security staff were killed, 106 civilians suffered head injuries, and 12 the loss of a limb, while approximately 8,163 reported less severe injuries. Throughout the demonstrations, 4,900 protesters were taken into police custody (Ete & Taştan, 2013). The psychological effects of the physical and psychological violence inflicted, however, have not been explored comprehensively. Violent acts throughout the demonstrations have been described as components of a human-made disaster (Kaptanoğlu, 2013). The complexity of state violence, which includes human rights violations, is related to its political context and consequences. The body of literature on human rights violations in Turkey (e.g., Başoğlu et al., 1994, 1997; Paker, 2000) has taken the contexts into account, in terms of the visibility of the violence to which protesters were exposed.

Among the literature, no study has yet looked at the psychological effects of the violence during the Gezi Park demonstrations. In addition, studies that investigated violence and took both PTSD and PTG into account are very scarce. Overall, this study investigates the Gezi Park demonstrations in terms of psychological trauma. Its aim is to gather information on individuals’ psychological conditions in large-scale potentially traumatic events. The current work adopts the following research questions: (1) Does political activism and psychological preparedness have effects on violence-induced posttraumatic stress levels (PTSL)? (2) Among the demonstrators, after statistically controlling for traumatic experience levels before and after the demonstrations and psychological preparedness for violence, do violence exposure levels, total amount of time spent at the demonstrations, perceived social support, and world assumptions predict PTSL? (3) Among the demonstrators, after statistically controlling for traumatic experience levels before and after the demonstrations and psychological preparedness for violence, do violence exposure levels, total amount of time spent at the demonstrations, perceived social support, and world assumptions predict PTG?

## Method

### Participants

A total of 516 participants completed the study, but 2 of the participants declared that they had not attended the demonstrations and were excluded from the study. Thus, the sample of the study consisted of 514 participants aged between 17 and 60 years (*M*_age_ = 27.80, *SD* = 7.50). There were 316 (61.5%) female and 196 (38.1%) male participants. Two participants did not respond to the gender item. At the time of the study, 234 (45.5%) of the participants lived in Ankara, 166 (32.3%) in Istanbul, 25 (4.9%) in Izmir, 13 (2.5%) in Eskişehir, 5 (1%) in Bursa, 14 (2.7%) abroad, and 57 (11.1%) in different parts of Turkey. In terms of education levels, 125 (24.3%) of the participants had completed a master’s degree or higher, 281 (54.7%) a bachelor’s degree, 105 (20.4%) high school, 2 (0.4%) middle school, and 1 (0.2%) participant had completed no school but was literate.

### Procedure

The Ethics Board of the Hacettepe University Senate evaluated and approved the study. Data were gathered through an online survey system by snowball sampling. To reach the people who could have possibly participated in the Gezi Park demonstrations, social media channels, various e-mail groups, political gatherings/groups/parties/solidarity networks, and post-demonstrations groups were contacted. Anonymity was guaranteed for participants in all phases of the study. The participants, who agreed to participate voluntarily and declared this by signing the voluntary participation form, filled out the demographic information form followed by the event information form and the scales. The total amount of time needed to complete the entire study was about 15 minutes; however, no time limit was established. No question could be skipped. The last window included a brief appreciation message for participating in the study.

### Measures

#### Demographic Information Form

To obtain a detailed profile of the participants, a comprehensive demographic information form was designed. In this form, participants were asked to state their age, gender, marital status, education level, occupation, residence, people they lived with (household sharing), previous psychiatric diagnoses, physical illness history, and whether they had a paid job. The main aim of asking this information was to see if there was a certain pattern of demographic features belonging to the demonstrators. However, no unique pattern was found and the characteristics was parallel with the commonsense opinion that the demonstrators were young adults with education level above the average, exhibiting no gender difference.

#### Event Information Form

The event information form was created to gather event-specific information. The participants were asked about the total amount of time they had spent at the demonstrations and the frequency of their participation in the demonstrations, as well as their behavior after police intervention. Only the former was used due to latter two not working properly as if the questions were not operationalized to cover the concepts. Throughout the literature, no scale was found to measure political activism and psychological preparedness for violence directly. In studies that dealt with psychological trauma and political activism, political activism was addressed within the demographics form with event-specific questions (Başoğlu et al., 1994; Kanagaratnam, Raundalen, & Asbjørnsen, 2005; Paker, 2000). In the present work, political activism and psychological preparedness for violence were approached in a similar way but as a distinct section, using an event information form. Participants were asked about their experience being in police custody, under arrest, in prison, or tortured for political reasons (1) before the Gezi Park demonstrations and (2) during the demonstrations. The participants were also asked to evaluate themselves in terms of political activity (1) before the Gezi Park demonstrations and (2) during the demonstrations. Moreover, participants were asked about their preparedness for the violence they faced during the demonstrations. At the end of this form, additionally, the first part of the Post-Traumatic Stress Diagnostic Scale (PTSDS) was added (Foa, Cashman, Jaycox, & Perry, 1997). This part identified traumatic event types (natural disaster, war, accident, etc.) as well as other possible traumatic events that participants might have gone through after the demonstrations.

#### Stress Symptoms Sub-Scale of the PTSDS

The scale was developed by Foa et al. (1997) to identify post-traumatic stress symptom levels in accordance with DSM-IV. The scale consists of 17 Likert-type items. Total scores range between 0 and 51; with higher scores indicating higher symptom levels. The internal consistency of the original scale was Cronbach’s α = .92, and the test-retest validity coefficient for items was .83 (Foa et al., 1997). Isıklı (2006) completed a Turkish adaptation of the scale, and the Cronbach’s alpha obtained for the total scale was .93. For this study, Cronbach’s alpha for the total scale was .91, and the Cronbach’s alpha for the “re-experiencing,” “avoidance,” and “hyperarousal” sub-factors was .83, .79 and .86, respectively.

#### PTG Inventory

Tedeschi and Calhoun developed the scale in 1996, with the aim of identifying growth in the aftermath of a traumatic event (Tedeschi & Calhoun, 1996). The scale consists of 21 items scored between 0 and 6. Total scores range between 0 and 105, with higher scores indicating a higher amount of growth. In the original study, internal consistency for the entire scale was α = .90, whereas for the sub-scales, it ranged between α = .67 and α = .85. The test-retest validity correlation coefficient was .71. Dürü developed a Turkish adaptation of the scale in 2006 (Dürü, 2006). The internal consistency coefficient obtained was Cronbach’s α = .93. For the present study, the internal consistency coefficient for the total scale was Cronbach’s α = .95. The internal consistency coefficients for the sub-factors obtained were Cronbach’s α = .87 for “relating to others,” α = .85 for “new possibilities,” α = .83 for “personal strength,” α = .90 for “spiritual change,” and α = .63 for “appreciation of life.”

#### Multidimensional Scale of Perceived Social Support

Zimet, Dahlem, Zimet, and Farley (1998) developed the scale in 1998 to measure perceived social support from three different sources: family, friends, and significant others. The Likert-type scale consists of 12 items scored between 1 and 7. Scores range between 12 and 84, with higher scores representing higher perceived social support. In the original study, the internal consistency coefficient for the “family” subscale was Cronbach’s α = .91, for “friends,” α = .87, and for “significant others,” α = .85 (Zimet, Dahlem, Zimet, & Farley, 1988). Eker and Arkar developed a Turkish adaptation of the scale in 1995, which they revised in 2001, to address the cultural material of the scale (Eker & Arkar, 1995; Eker, Arkar & Yaldız, 2001). In different samples, the internal consistency coefficients for the total scale were found to be between Cronbach’s α = .80 and α = .95, and for the subscales, between Cronbach’s α = .78 and α = .91. For the current study, the internal consistency coefficient for the total scale was Cronbach’s α = .93, for the “family” subscale, Cronbach’s α = .93, for “friends,” Cronbach’s α = .95, and for “significant others,” Cronbach’s α = .96.

In the current study, dimensions of social support was taken separately with the aim of understanding specific sources of support that might be more salient in collective violence experience.

#### The World Assumptions Scale

The scale was developed by Janoff-Bulman in 1989 with the purpose of identifying and measuring the effects of traumatic stress on fundamental assumptions. The total scale is composed of 32 items scored between 1 and 6 (Janoff-Bulman, 1989). The original form consisted of seven sub-scales: benevolence of the world, randomness, justice, controllability, self-worth, self-control, and luck. Yılmaz prepared a Turkish adaptation of the test in 2008. The adapted version consists of 31 items under six subscales: benevolence, randomness, justice, control, self-worth, and luck. The internal consistency coefficients for the total scale were found to be Cronbach’s α = .70, and for the subscales, .58, .59, .57, .47, .13, and .85, respectively (Yılmaz, 2008). For this study, the total scale internal consistency coefficient was Cronbach’s α = .84, whereas those for the subscales were .88, .37, .83, .68, .61, and .90, respectively.

In the current study, sub-scales of WAS was taken individually throughout the analysis. The reason behind doing so was that, having assumed the world assumptions as a whole would be shattered, each dimension was thought to be referring to a different concept that might be emphasizing on a different aspect of world assumptions specific to the event. Thus, it was done to understand the specific world assumptions that might be shattered with the experience of violence during political demonstrations.

### Data Analysis

The data were analyzed using SPSS (Version 22.0). The dataset gathered from the forms and the scales was examined in terms of missing values and normality, and the characteristics of the variables met the assumptions of normality. As the first step of analysis, the frequency values of demographics were explored.

The traumatic events before and after the demonstrations might have had a confounding effect; thus the aim was to control possible effects statistically by creating two control variables. The first control variable, *traumatic experience levels before the demonstrations*, constituted having been in police custody, under arrest, in prison, or tortured for political reasons before the demonstrations, with an index range between 0 and 4 in yes/no statement. The second control variable, *traumatic experience levels after the demonstrations*, consisted of the nine-item from the first part of the PTSDS, each item adding 1 point to the total. This index identified any traumas (natural disaster, physical assault, sexual assault, fatal disease, etc.) the participants experienced after the demonstrations to control for the confounding effects.

For the political activism variable, participants were asked to mark the best-fitting option (being an activist, being a member of a political group but not being politically active, being politically active but not being member of a political group and being politically inactive). Before starting the analysis, the first two answers were merged due to both groups being parts of a political group and ideology. New groups were activists (members of a political group), politically active (not member of a political group) and politically inactive.

The open-ended question regarding having experienced violence (16 sub-dimensions), placed in the event information form, and the question on having been in custody, under arrest, in prison, or tortured for political reasons during the demonstrations (4 sub-dimensions) were combined to form the total *violence index.* The violence index had 20 sub-dimensions, 16 of which were derived from the open-ended question asking to explain the experiences of violence, to form groups of different violence types depending on the frequencies. As the first step, the responses were read by the author and searched for the general violence categories defined by World Health Organization: physical, psychological, sexual, emotional. Regardless of the type, existence of each categories added 1 point. Also, research on collective violence emphasized possible existence of economic violence so it was added as a category. Rest of the violence types were derived from the participants’ experiences (verbal abuse, assault, pepper gas, pressure water, beaten with police baton, demonstrator violence, etc.) The violence index scores ranged between 0 and 20, with the presence of each violence sub-scale valued 1, and the absence, 0. After the standardization of data, the relationship each of the independent variables with the dependent variables (post-traumatic stress symptom levels [PTSSL] and PTG) was examined through Pearson moment product correlation coefficient analysis.

For the preparedness for violence variable, participants were directly asked psychologically how prepared would they define themselves to the violence witnessed. The 5 options were well-prepared, prepared, neither prepared nor unprepared, not prepared and not prepared at all.

The total amount of time spent at the demonstrations was asked directly to the participants. They were expected to answer in days and hours; and the maximum hours one can participate in the demonstrations was 6 hours a day where the duration of demonstrations was taken as 20 days (maximum number of days one can participate in the demonstrations). So, the maximum amount of time that could be spent at the demonstrations were 120 hours.

In the following step, to examine the effects of political activism and psychological preparedness for violence on PTSSL, a 3 (activist / politically active / politically inactive) x 5 (well-prepared / prepared / neither prepared nor unprepared / not prepared / not prepared at all) analysis of variance (ANOVA) was conducted. Lastly, hierarchical regression analyses were carried out to identify the predictors of PTSSL and PTG. As psychological preparedness was found to have a significant effect on PTSSL, it was included as a control variable in the first step of the regression analysis together with the pre-event and post-event indices.

## Results

### Effects of Political Activism and Psychological Preparedness for Violence on PTSSL

No significant interaction effect between political activism and psychological preparedness for violence was found, *F*(8, 514) = 1.06, *p* = .388, $\eta_{p}^{2}$ = .02; Table 1. However, the main interaction effects for political activism, *F*(2, 514) = 3.31, *p* = .037, $\eta_{p}^{2}$ = .01, and psychological preparedness for violence, *F*(4, 514) = 12.47, *p <* .001, $\eta_{p}^{2}$ = .09, on PTSSL were found to be significant. Between-group differences were examined through the Tukey test, using Bonferroni correction (Table 3). The PTSSL of the activist group (*M* = 32.8) were found to be significantly higher than that of the politically inactive group (*M* = 28.7). However, further analysis were carried through splitting the data in terms of political activism levels and on none of the variables did political activism indicate meaningful results. Even though the main effect of political activism was significant, with the data in hand, no further inference could be made. Thus, political activism variable was not included in the regression models.

**Table 1:** ANOVA Results of Political Activism and Psychological Preparedness for Violence on PTSSL

Variable	*n*	*M*	*SE*	ANOVA		
				*df*_1_, *df*_2_	*F*	$\eta_{p}^{2}$
Political activism				2, 499	3.31*	.01
Activist	115	32.8_a_	1.3			
Politically active	216	30.9_ab_	0.8			
Politically inactive	183	28.7_b_	0.9			
Psychological preparedness for violence				4, 499	12.47**	.09
Well-prepared	50	24.1_a_	1.7			
Prepared	119	27.9_a_	0.9			
Neither prepared nor unprepared	167	29.2_a_	0.8			
Not prepared	114	34.0_b_	1.2			
Not prepared at all	64	38.7_b_	1.9			

*Note*. Different subscripts refer to the significant differences between means.

*p < .05. **p < .001.

The main interaction effect for psychological preparedness for violence on PTSSL was found to be significantly lower for the “well-prepared” (*M* = 24.1) and prepared groups compared with the “not prepared” (*M* = 34.0) and “not prepared at all” (*M* = 38.7) groups, separately. Similarly, the PTSSL of the “neither prepared nor unprepared” (*M* = 29.2) group were found to be significantly lower compared with the “not prepared” (*M* = 34.0) and “not prepared at all” (*M* = 38.7) groups.

### Analysis of Relationship Between Variables

The relationship between independent and dependent variables was examined through Pearson moment product correlation coefficient analysis (Table 2). PTSSL and violence exposure level (*r* = 18, *p* < .01) and the randomness sub-scale of WAS (*r* = .17, *p* < .01) were positively correlated; whereas the self-worth sub-scale of WAS (*r* = -.22, *p* < .01) was negatively correlated. PTGL were found to be positively correlated with violence exposure levels (*r* = .20, *p* < .01), total amount of time spent at the demonstrations (*r* = .16, *p* < .01), and the following WAS sub-scales: benevolence (*r* = .21, *p* < .01), luck (*r* = .17, *p* < .01), justice (*r* = .19, *p* < .01), randomness (*r* = .10, *p* < .05), and control (*r* = .14, *p* < .01).

**Table 2:** Correlation Coefficients of Relationship between PTSSL/PTGL and Other Variables

Variable	PTSSL	PTGL
Violence exposure level	.18**	.20**
Total time	.08	.16**
Sub-scales of MSPSS		
Family	-.04	.06
Significant others	-.07	.02
Friends	-.06	.03
Sub-scales of WAS		
Benevolence	-.04	.21**
Justice	.04	.19**
Luck	.01	.17**
Randomness	.17**	.10*
Self-worth	-.22**	-.04
Control	.09*	.14**

*Note*. PTSSL = Predictors of post-traumatic stress symptom levels. PTGL = Post-traumatic growth levels.

*p < .05. **p < .01.

### Hierarchical Regression Analysis for the Predictors of PTSSL

In the first step of the regression equation, traumatic experience levels before and after the demonstrations and psychological preparedness for violence were entered as control variables (Table 3). In the second step, violence exposure levels and total amount of time spent at the demonstrations were entered. Lastly, sub-scales of MSPSS and WAS were entered as separate blocks into the regression equation.

Control variables “traumatic experience levels before the demonstrations”, β = -0.03, *t*(514) = -0.64, *p* = .526, “traumatic experience levels after the demonstrations”, β = 0.13, *t*(514) = 2.99, *p* = .003, and “psychological preparedness”, β = 0.30, *t*(514) = 6.87, *p* < .001 contributed significantly to the regression model and accounted for 11% of the variation, Δ*F*(3, 491) = 20.32, *p* < .001. In the second step, only “violence exposure levels”, β = 0.21, *t*(514) = 4.72, *p* < .001, showed a predictor value on PTSSL and raised the total variance to 15% with an impact of 5%, Δ*F*(1, 490) = 20.61, *p* < .001. The “significant others” sub-scale had a predictor value; the total variance explained was raised to 16%, Δ*F*(1, 489) = 6.94, *p* < .001. Lastly, from the WAS sub-scales, “randomness”, β = 0.17, *t*(514) = 3.53, *p* < .001, and “self-worth”, β = -0.15, *t*(514) = -3.51, *p* < .001, raised the variance to 21%, Δ*F*(1, 488) = 16.44, *p* < .001; Δ*F*(1, 487) = 12.21, *p* < .001, respectively.

**Table 3:** Results of the Hierarchical Regression Analysis on Scores From the Stress Symptoms Sub-Scale of the Posttraumatic Stress Diagnostic Scale

Variable	β	*t*	*R*^2^	Δ*R*^2^	Δ*F*
Step 1					
TEL before	-0.03	-0.64			
TEL after	0.13	2.99			
Psychological preparedness	0.30	6.87	.11	.11	20.32*
Step 2					
Violence exposure level	0.21	4.72*	.15	.04	20.61*
Step 3					
Significant others	-0.11	-2.11*	.16	.01	6.94*
Step 4					
Randomness	0.17	3.53*	.19	.03	16.44*
Self-worth	-0.15	-3.51*	.21	.02	12.21*

*Note.* TEL = Total exposure level.

*p < .001.

### Hierarchical Regression Analysis for the Predictors of PTG

The same procedure was followed as in the previous regression analysis. Entered as control variables in the first step, “traumatic experience levels before the demonstrations”, β = -0.04, *t*(514) = -0.79, *p* = .430, “traumatic experience levels after the demonstrations”, β = 0.08, *t*(514) = 1.88, *p* = .061, and “psychological preparedness for violence”, β = 0.02, *t*(514) = 0.48, *p* = .632, accounted for 2% of the variation, Δ*F*(3, 491) = 3.41, *p* = .018. “Violence exposure levels,” included in the second step, were found to raise the variance to 4%, Δ*F*(1, 490) = 14.55, *p* < .001, whereas “total amount of time spent at the demonstrations”, β = 0.13, *t*(514) = 2.58, *p* = .010, was found to have 1% impact, raising total variance explained to 5%, Δ*F*(1, 489) = 7.33, *p* < .001. None of the sub-scales of MSPSS entered in the following step was found to have predictor power on PTG (Table 4).

**Table 4:** Results of the Hierarchical Regression Analysis on Scores From the Posttraumatic Growth Inventory

Variable	β	*t*	*R*^2^	Δ*R*^2^	Δ*F*
Step 1					
TEL before	-0.04	-0.79			
TEL after	0.08	1.88			
Psychological preparedness	0.02	0.48	.02	.02	3.41*
Step 2					
Violence exposure level	0.18	3.58**	.05	.03	14.55**
Total time	0.13	2.58**	.06	.01	7.33**
Step 3					
Benevolence	0.20	4.76**	.10	.04	22.46**
Luck	0.13	2.82**	.12	.01	8.13**
Justice	0.11	2.27*	.13	.01	4.72*

*Note.* TEL = Total exposure level.

*p < .05. **p < .01.

Lastly, “benevolence” (β = 0.20, *p* < .001), “luck” (β = 0.13, *p* < .001), and “justice” (β = 0.11, *p* < .001) were found to have predictor value and accounted for 6% of the variance; raising the total variance to 11%, Δ*F*(1, 488) = 22.46, *p* < .001; Δ*F*(1, 487) = 8.13, *p* < .001; Δ*F*(1, 486) = 4.72, *p* < .001, respectively.

## Discussion

The study found no significant interaction effect between political activism and psychological preparedness for violence. However, the main effects for political activism and psychological preparedness for violence on PTSSL were significant. Hierarchical regression analyses revealed that after statistically controlling for traumatic experience levels before and after the demonstrations and psychological preparedness for violence, (1) PTSSL were predicted by violence exposure levels, the MSPSS sub-scale “significant others,” and the WAS sub-scales “randomness” and “self-worth”; (2) PTGL were predicted by violence exposure levels, time spent at the demonstrations, and the WAS sub-scales “benevolence”, “luck,” and “justice.”

In the current study, results on political activism and psychological preparedness for violence, the two variables frequently studied together were found to have a significant effect on PTSSL individually but not together, conflicting with previous studies (Başoğlu et al., 1994, 1997; Paker, 2000). Previous research proposed that possible repetitive traumatic stress throughout political struggles, political activists might acquire immunity to traumatic stress (Başoğlu et al., 1994). However, in the present study, the opposite tendency of this immunity against stress was found: higher PTSSL were seen in the politically active group than the inactive group. This finding may be the result of the politically active group’s higher exposure to violence before and after demonstrations. Prior studies on political activism studied the effects of being in police custody or jail for political reasons (Başoğlu et al., 1994, 1997; Paker, 2000). However, state violence during the Gezi Park demonstrations took place at a more public sphere than the violence in prison or under surveillance. We assume that this public nature brought about higher social support, like social sharing and cooperation. In addition, during the demonstrations, deterrents were largely used as tools for violence, whereas more systematic ways of violence are applied in prisons and under surveillance (Paker, 2000). Thus, event-specific factors seemed to affect the results and explain the failure in covering some concepts. For the political activism variable, it is possible that the operationalization and measuring has failed to grasp the concept. It is highly recommended for future studies to well-operationalize and standardize the concept and measurement of political activism with its all dimensions. This study has mostly focused on the ‘being part of a political group and ideology’ but it is possible to say that there was missing aspects and levels of it.

Meanwhile, as psychological preparedness for violence tended to increase, PTSD symptoms tended to decrease. Previous research proposed that psychological preparedness relates to the severity of distress during torture and other severe psychological issues. Thus, psychological preparedness functions like a “vaccination” (Başoğlu et al., 1997). The present finding on preparedness might confirm this “vaccination” effect, although the phenomenon needs to be understood more profoundly. Findings on the predictors of PTSSL showed that as the violence index scores increased, PTSSL increased. As the violence index contains many physical violence statements, literature (North et al., 1999; Paker, Paker, & Yüksel, 1992) on violence experiences indicated that physical damage would result in higher PTSSL.

The MSPSS sub-scale “significant others” was found to be another predictor of PTSSL; higher social support perceived from significant others related with lower PTSSL. This result may relate with factors specific to the sample, mainly age. The participants were mostly in their early adulthood (*M*_age_ = 27.80, *SD* = 7.50), in which the need for close relationships is significant, as pointed out in studies (Erikson & Erikson, 1998). The WAS sub-scales “randomness” and “self-worth” likewise predicted PTSSL. This finding may be related with the attributions on the meaningfulness of the world, which is encompassed in people’s beliefs on the distribution of outcomes (Janoff-Bulman, 1989). A sub-dimension of this belief is randomness, in which people believe that specific events and consequences happen randomly; people with strong randomness beliefs have the belief of having no control over negative events (Janoff-Bulman, 1989). Viewed from this perspective, the Gezi Park demonstrations were a collective movement against the political authority and action to change the perceived injustice (Doğan, 2014); thus, demonstrators with high randomness belief and low PTSSL may have less belief in their potential to change the injustice. Moreover, the “self-worth” sub-scale indicates people’s beliefs about themselves; namely, to what extent they see themselves as good, moral, and worthy, how properly they behave, and to what extent they are able to protect themselves from bad luck (Janoff-Bulman, 1989). This study showed that a decrease in demonstrators’ belief of self-worth related with PTSSL.

Elevated violence exposure indicating PTG was parallel with the literature. PTG and PTSSL could be present simultaneously, with some amount of stress necessary for growth (Calhoun & Tedeschi, 1998). Meanwhile, the total amount of time spent at demonstrations predicting growth may be related to the severity of the traumatic event. The total amount of time spent at the demonstrations predicted PTG, but not PTSSL, which might be explained by the findings that PTG occurs after a long period (Tennen & Affleck, 1998). Those with present PTG findings might have suffered from elevated PTSSL in the past, indicating that PTG is not the result of post-traumatic stress but the process occurring over time.

The first sub-scale of the WAS predicting PTG was benevolence of the world. According to benevolence assumption, people are good, kind, and helpful, and the world is a good and safe place (Janoff-Bulman, 1989). Findings on attenuated benevolence assumption predicting PTG may point to the shattered assumptions owing to trauma being rebuilt.

Another sub-scale was luck found to predict PTG, in which the world is thought to work according to chance and only the lucky people could get good outcomes (Janoff-Bulman, 1989). This sub-scale predicting PTG may be related with people’s attributions on physical injury; distribution of the physical violence being attributed to luck.

Lastly, the justice sub-scale was found to predict PTG. According to this assumption, people get what they deserve and deserve what they get (Janoff-Bulman, 1989). The justice assumption on the distribution of outcomes includes giving an answer to bad outcomes. Thus, the findings may relate with giving meaning to the traumatic event: who experiences violence to what extent.

Moreover, demonstrators’ that participated in the study had the mean age of 27.8 overlapping the previous research (TİHK, 2014; Ete & Taştan, 2013). As widely discussed in the days of demonstrations, these findings might be indicating to a generation’s political behavior. These young cohorts seemed to express their political demands and attitudes in a more expressive way than the older cohorts. It is a matter of interest to explore this difference through a longitudinal method to see if this is a result of age or generational difference in terms of political attitudes and behaviors.

The psychological viewpoint on political traumas is based on contextual assumptions (Montiel, 2000). The first assumption is on traumatic events being intense but short. In the present study, when referring to the experience of violence, participants mostly mentioned a short time frame in which the experience was intense. The second assumption is on the effects of the political context on traumatic event survivors; it was assumed that the political context was evil itself according to the survivors (Montiel, 2000). During the demonstrations, the political context might have been perceived as so evil that despite the massive violence, it took almost one and a half months for street demonstrations to subside (Ete & Taştan, 2013). The last assumption is on the relationship between survivors and the political context (Montiel, 2000). As mentioned by Suedfeld (1997), survivors are not only victims, but also have the potential to be modifiers of the political context. When studying trauma, especially political trauma, the contextual perspective needs to be considered as well. Being active subjects in the political context might have recuperative effects and might relate with PTG. Humor, a collective way of coping during demonstrations, is thought to have a healing role. In this sense, Garrick (2006) mentioned that humor had healing effects in the aftermath of trauma.

### Limitations

Firstly, when the data collection of the present study was completed, almost two years had passed since the Gezi Park demonstrations. Although subjects like war, migration, and torture are frequently examined retrospectively throughout literature, the current results might have been influenced with the elapsed time. Meanwhile, as previously mentioned, for PTG to be observed, the passing of time is seen to be necessary, making longitudinal studies or repeated measures studies more reliable when studying PTSSL and PTG together.

Although the study mostly relied on analysis from quantitative data, the qualitative data gathered were rich and contributed significantly to the study. Approaching multidimensional subjects both through quantitative and qualitative forms could make the information richer. However, the standardization of the qualitative data is challenging. When working with political activism and psychological preparedness, it is recommended that data be collected in a way that is detailed, systematic, and suitable to the nature of the trauma.

During the Gezi Park demonstrations, many people were exposed to violence through the media or were secondary victims in some other ways. Focusing on the secondary traumatization aspect might be enlightening. As Becker (1995) mentioned, the notion of PTSD might have deficiencies in grasping traumas within the sociopolitical context. In this sense, taken as “post” traumatic, PTSD assumes trauma occurring at a certain time interval with a beginning and an end; however, in the case of many traumatic events, trauma might be perpetual. Moreover, the tendency to diagnose survivors of traumas with a “disorder” has been criticized, as the symptoms of the survivors may be the results of fundamental ethics violations (Becker, 1995). Thus, studying the effects of the traumatic event solely through PTSD might engender a narrow understanding of the phenomenon. In addition, it has been suggested that reactions to the traumatic event have a culture-specific aspect (Shannon et al., 2015). PTSD studies can grasp the symptoms beyond the cultural factors; however, culture-prone dimensions might be left out.

## Author Note

This article was written within the scope of the Master's thesis of the first author (Yeniada, 2015).
